# On Paracentesis Thoracis

**Published:** 1845-01

**Authors:** 


### Paracentesis Thoracis. (Guy's Hospital Reports.)

In our 81st number (July 44) we gave a copious analysis of the report of Messrs. Hughes and Cock, on the above subject. In the October report from Guy's Hospital, Dr. Hughes has appended some additional cases, of which we shall introduce here a brief account.

*Case* 1.—A man, aged 44, thin and spare, a fish and fruitseller in the streets, had been attacked, a month previous to admission, as an out-patient, with pain in right side, cough, and dyspnœa, for which medicines were prescribed without benefit. He was afterwards admitted into hospital. The right side ribs did not rise in inspiration—could lie in any position in bed. The physical signs were entire dulness throughout the right side—respiration distant and tubular—no enlargement of side, or intercostal protrusion—no enlargement of liver, or displacement of heart. The exploration shewed the presence of considerable fluid. Mercury, diuretics, blisters, &c. having failed, the trochar was introduced, and 30 ounces of clear fluid drawn off, not coagulable but by heat. Ten days afterwards, the operation was repeated, and twelve ounces abstracted. The diuretics were now pushed a good deal, and mercury was exhibited.

"He was afterwards again put under the influence of mercury; and being now free from any cough or disturbance of the respiration; being able to lie down with ease; to walk about without inconvenience; to eat, drink, and sleep well; and his principal complaints being of flatulence and palpitation, probably resulting from dyspepsia; the respiration having returned in a great degree in even the lower part of the right side posteriorly, and to some, though a less extent and degree anteriorly, though the whole side was still very dull on percussion, and the voice shrill throughout its greater portion; he left the hospital August 13th, with the belief that he would be improved by air and exercise, and with the promise that he should return if he were not so well. Up to the present date, September 13, he has not since been seen or heard of." 357.

*Case* 2.—A man, aged 34, admitted under Dr. Addison, June 25, 1844. The patient had been a medical practitioner, and first fell ill in Nov. 1842, in New South Wales, with cough, expectoration, emaciation, dyspnœa, &c. In February, 1843, he had hæmoptysis. In July he sailed for England, and arrived in the latter end of November, when the right side of the chest was somewhat bulged out. On admission by Dr. Addison—

"He had a pulse of 120, considerable dyspnœa, and an entire incapability of lying on the left side, or on the back. He had very little cough, accompanied with trifling mucous expectoration, a clean tongue, a good appetite, and was free from nocturnal perspirations. The right side of the chest was then very dull on percussion both behind and before, including the entire length and breadth of the sternum: it was somewhat misshapen, and measured about half an inch more than the left side. Tubular breathing was heard throughout the whole of the right side, excepting immediately below the clavicle and about the spine of the scapula, where some harsh respiration, together with fine but loose mucous rattle, was indistinctly audible. The voice was tolerably resonant over the whole of this side of the chest, and possessed a shrill ægophonic character below the angle of the scapula. The impulse of the heart was felt more than an inch nearer to the axilla than in the healthy state. *The liver could not be felt below the ribs, nor was any sulcus apparent, though both were sought after with some interest.* There existed no general elevation or bulging of the intercostal spaces; but a soft, flat, fluctuating tumor, nearly the size of the palm of the hand, which increased upon coughing, was observed to overlie, and to proceed from between, the sixth and seventh ribs.

"On the left side, dullness on percussion, increased resonance of the voice, and imperfect respiratory murmur, existed in the mammary and hypochondriac regions; and just above and to the outer side of the nipple, over a space the size of a crown-piece, increased dullness on percussion, tubular breathing, imperfect pectoriloquism, and gurgling rattle, were clearly distinguished. The other parts of the left side presented nothing abnormal; and the heart, with the exception of its being displaced, appeared healthy." 259.

On exploration, the left cavity of the pleura was found to contain much fluid, and the trochar was introduced, when 24 ounces of fluid were evacuated by Mr. Cock, in a full stream, 27th June. From imprudent exertion, some hæmoptysis was occasioned, and was checked by super-acetate of lead and hyosciamus. He was ordered iodide of potassium with sarsaparilla, and sent into the country. July 15th. Returned, and paracentesis was again performed on the 16th, by Mr. Cock, and 36 ounces of fluid withdrawn in a full stream, with great relief to the breathing. Pil. hyd., ext. hyosciam. and liq. potass. were ordered. He left the hospital again, and returned on the 30th July.

" A very gratifying change was now apparent. The girth of the chest measured at least two inches less than formerly, the decrease being nearly equally divided between the two sides. He could lie and sleep, without difficulty, on the back and left side; his health had much improved; his tongue was clean; and his breathing comparatively easy. The entire sternum remained resonant upon percussion; and the upper part of the right side, nearly as low as the nipple, had regained its sonorousness. The vesicular murmur was audible in the right side posteriorly, and, though mixed with mucous rattle, and harsh in character, as low as the nipple anteriorly. The right side expanded, upon inspiration, nearly as fully as the left; the dullness, gurgling, and resonance of the voice above the left nipple had almost entirely disappeared, the particular space being indicated principally by a little muco-crepitating rattle; and the impulse of the heart was felt in nearly its normal site." 361.

He left the hospital soon afterwards.

*Case* 3.—A lad, aged 18, was admitted on the 31st July, 1844. Became affected with pain in the chest eight months previously, and in March last was obliged to give up work as a painter. Upon admission he had pain in the left side—pallid countenance—tongue clean—bowels irregular—respiration 42—pulse 120, feeble.

" The physical signs presented, on examining the chest, were, dullness on percussion as high as the fourth rib on the left and the sixth rib on the right side anteriorly, and at the angle of the scapula on the left side posteriorly. The respiratory murmur was defective, or distant on both sides at the lower, and puerile on both at the upper parts of the chest. Increased resonance of the voice existed over the whole chest, except at the inferior part of the left side before and behind. A little pleuritic rubbing or creaking was heard, together with shrill bronchophony, along the outer edge of the lower part of the scapula. The apex of the heart was felt most distinctly to strike the parietes between the fourth and fifth ribs, below and rather to the inner side of the nipple: its impulse was feeble; its sounds were triple. There appeared, indeed, to be a double first sound. The rhythm may be thus represented—tic-tic—tac; tic-tic—tac. Its abnormal character seemed to be probably connected with effusion between the pleura and pericardium." 363.

On the 1st of August the explorator was introduced, and a few drachms of clear fluid followed. Opium and quinine—liq. potassæ. On the 14th August another operation was performed, and seventeen ounces were drawn off. A flannel bandage was applied. On the 20th August,

" The chest measured one and a half inch less than before, almost the whole of which was dependent on the decrease of the left side. He now lay upon the right or left side with equal facility; had no cough; and had much improved both in flesh and appearance since his admission. His respirations were 36 in the minute, and his pulse 112, but still very feeble. This day, Sept. 7th, he has been again examined. He makes no complaint, excepting of the soreness of a blister recently applied: the tongue is clean, the respirations 22 in the minute, but the pulse still numbers 112 to 120, and is very feeble. The left side is throughout resonant upon percussion, and the respiratory murmur can be heard in every part, the only abnormal circumstance discovered being a little pleuritic creaking, resulting from adhesions. The treble beat of the heart, also, has disappeared. As he seems now to be suffering more from debility and confinement than from any remains of his complaint, he has been advised to go into the country for change of air, on Monday next, the ninth instant, with a desire that he shall present himself for examination upon his return to the neighbourhood of town." 364.

These and the cases previously reported, shew that paracentesis seldom produces any inconvenience, and often gives great relief—perhaps preserves the life of the patient. The authors of this remedial means richly deserve the thanks of their brethren at large, not only for their courage and candour, but for rescuing such valuable facts from the dark records of hospital books, where important information too often lies in oblivion. We hope the time is fast approaching when these treasures of practical knowledge will be regularly laid open to the public.

